# Chemotherapy sensitivity testing on ovarian cancer cells isolated from malignant ascites

**DOI:** 10.18632/oncotarget.27827

**Published:** 2020-12-08

**Authors:** Judith E. den Ouden, Guido J.R. Zaman, Jelle Dylus, Antoon M. van Doornmalen, Winfried R. Mulder, Yvonne Grobben, Wilhelmina E. van Riel, Joanne A. de Hullu, Rogier C. Buijsman, Anne M. van Altena

**Affiliations:** ^1^Radboud Institute for Health Sciences, Radboud University Medical Center, Obstetrics and Gynecology, Nijmegen, The Netherlands; ^2^Netherlands Translational Research Center B.V., Oss, The Netherlands

**Keywords:** ovarian cancer, chemotherapy sensitivity, prediction, ascites, proliferation assays

## Abstract

Background: In epithelial ovarian cancer (EOC), 15–20% of the tumors do not respond to first-line chemotherapy (paclitaxel with platinum-based therapy), and in recurrences this number increases. Our aim is to determine the feasibility of cell proliferation assays of tumor cells isolated from malignant ascites to predict *in vitro* chemotherapy sensitivity, and to correlate these results with clinical outcome.

Materials and Methods: Ascites was collected from twenty women with advanced EOC. Cell samples were enriched for tumor cells and EOC origin was confirmed by intracellular staining of CK7, surface staining of CA125 and EpCAM, and *HE4* gene expression. *In vitro* sensitivity to chemotherapy was determined in cell proliferation assays using intracellular ATP content as an indirect measure of cell number. *In vitro* drug response was quantified by calculation of the drug concentration at which cell growth was inhibited with 50%. Clinical outcome was determined using post-treatment CA125 level.

Results: Cell samples of twenty patients were collected, of which three samples that failed to proliferate were excluded in the analysis (15%). Three other samples were excluded, because clinical outcome could not be determined correctly. In twelve of the fourteen remaining cases (86%) *in vitro* drug sensitivity and clinical outcome corresponded, while in two samples (14%) there was no correspondence.

Conclusions: Our study demonstrates the feasibility of drug sensitivity tests using tumor cells isolated from ascites of advanced EOC patients. Larger observational studies are required to confirm the correlation between the *in vitro* sensitivity and clinical outcome.

## INTRODUCTION

Epithelial ovarian cancer (EOC) is the most lethal gynecologic malignancy worldwide [1]. Most EOC patients (70%) are diagnosed in an advanced stage, *i.e.,* International Federation of Gynecology and Obstetrics (FIGO) stage IIb – IV. The prognosis of advanced stage EOC is poor, with a 5-year survival rate of 20–30% [1–3].

Standard first-line treatment for advanced EOC consists of a combination of a debulking surgery and chemotherapy, with paclitaxel and a platinum-based compound administered either intravenously (IV) or intraperitoneally (IP) [4]. Despite extensive treatment, the prognosis of ovarian cancer has only slightly improved over the last three decades. In approximately 15–20% of women with EOC the tumor does not respond to first-line chemotherapy, and in recurrences this number increases due to drug resistance [5]. Although differences in therapy response have been reported between different histological subtypes [6], histology is not taken into account when therapy is selected. However, ovarian cancers with *BRCA* germline and somatic mutations are more sensitive to platinum-based therapy and Poly (ADP ribose) polymerase 1 (PARP)-inhibitors, and have better overall survival [7, 8]. Approximately 10–20% of EOC tumors harbor germline mutations in either *BRCA1* or *BRCA2* [9, 10]. About 60–70% of the *BRCA* positive tumor tests are based on a germline mutation, the others are somatic mutations [10]. *BRCA* gene mutation status is a patient stratification marker for PARP inhibitors, but not for first line platinum-based chemotherapy [7–10].

There is a need of more tools and assays to predict the clinical response to chemotherapy in EOC patients [3]. Ideally, this would avoid suboptimal treatment and prevent delay of optimal treatment, and thus clinical deterioration, unnecessary side effects of inadequate chemotherapeutics, and high societal costs.

EOC patients often present with high volumes of malignant ascites, which is easily accessible, and is routinely collected for diagnostic purposes or relief of complaints. Ascites is potentially an excellent source for biomarker discovery [11]. Konecny and co-workers reported a significant decrease in progression-free and overall survival (PFS, OS) of patients tested to be resistant *in vitro* using ATP tumor chemosensitivity assays performed on tumor cells isolated from biopsies [12]. Other studies describe the use of biomarkers in ascites to predict responses to initial therapy [13–15], although there are currently no biomarkers that have been validated. Furthermore, there are no studies that describe the use of *in vitro* cell proliferation assays to predict response to primary treatment.

A more personalized, tumor-specific treatment for EOC patients would be of great value to improve therapeutic decision-making and clinical outcome. The aim of this study is to determine whether tumor cells isolated from ascites of EOC patients can be used to determine chemotherapy sensitivity by using *in vitro* proliferation assays.

## RESULTS

**Figure 1:**
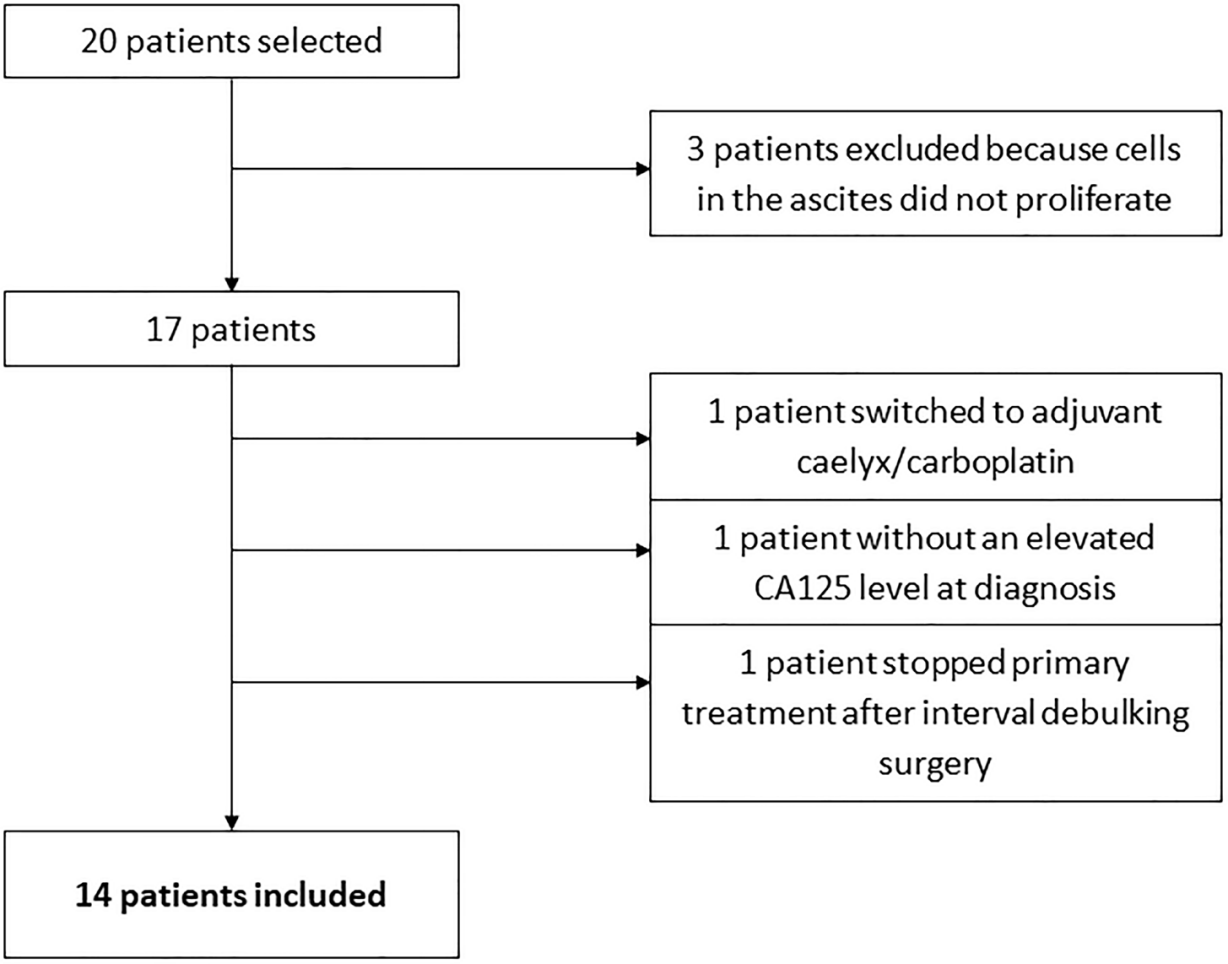
Flowchart of in- and exclusions.

We identified twenty patients diagnosed with EOC and presenting with ascites, who gave consent for the study. Three patient samples (ps.) (15%) were excluded, because cells isolated from the ascites did not proliferate *in vitro*. The clinical outcome of three other patients could not be determined correctly. One patient stopped primary treatment because she refused a relaparotomy for an anastomotic leakage after interval debulking surgery. The second patient did not have an elevated level of cancer antigen 125 (CA125) in serum at time of diagnosis. The third patient switched after interval debulking surgery to liposomal doxorubicin (Caelyx) plus carboplatin. These three patients were also excluded. Of the remaining fourteen patients included in the study, the median age at diagnosis of the patients was 62 years (range 50–71 years). The Karnofsky score at time of diagnosis for patients was between 70 and 90. See Table 1 for an overview of the clinicopathological data, and Figure 1 for an overview of in- and exclusions. All patients received carboplatin or cisplatin, and paclitaxel as a first-line intravenous (IV), or intraperitoneal (IP) chemotherapy treatment. Three patients received IP cisplatin, of which two finished the six cycles (ps. 1 and 6). One patient (ps. 3) also received IV carboplatin because IP-chemotherapy was stopped after one cycle.

**Table 1:** Patient characteristics

Median age in years	Frequency (*n* = 14)
	62 (range 50–71)
Primary origin of tumor	
Ovarian	4
Fallopian tubes	2
Adnexal	6
Primary peritoneal	1
Unknown	1
Karnofsky performance status	
70	2
80	5
90	6
Unknown	1
Treatment	
Primary debulking	7
Interval debulking	6
Only chemotherapy	1
Type of chemotherapy treatment	
Carboplatin + paclitaxel	11
Cisplatin + paclitaxel	2
Both carbo- and cisplatin + paclitaxel	1

**Figure 2:**
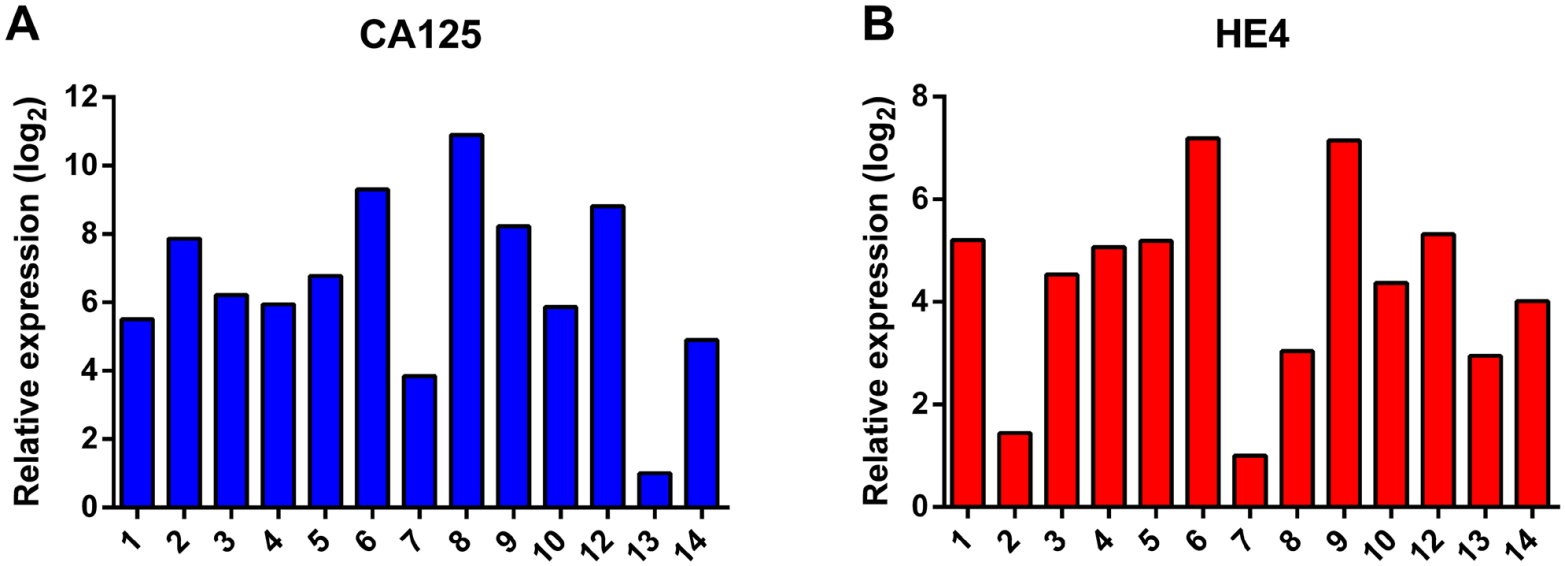
Analysis of the RNA expression of the ovarian cancer marker genes *CA125* (**A**) and *HE4* (**B**). Expression levels were normalized to the expression of the housekeeping gene β-actin (*ACTB*) and ribosomal protein S18 (*RPS18*).

**Figure 3:**
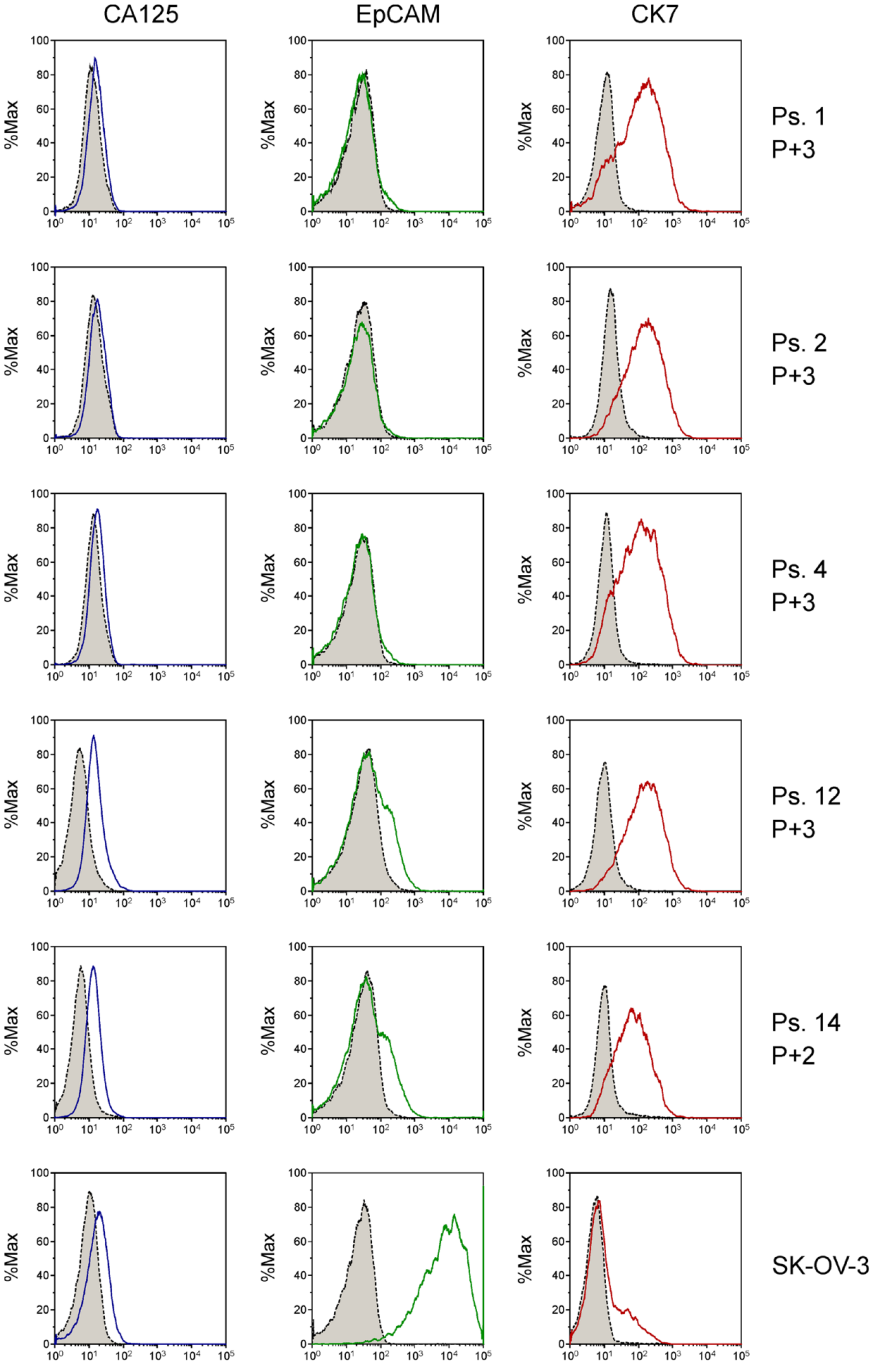
Analysis of tumor cell markers CA125, EpCAM and CK7 on primary patient-derived cell cultures by flow cytometry using fluorescently labeled antibodies. Grey-shaded peaks represent the staining with isotype control antibodies. Passage number (P) from collection of cells from ascites is indicated. The adenocarcinoma ovarian cancer cell line SK-OV-3 was analyzed for reference. Expression was quantified by determination of shift in fluorescence peaks (Table 3).

**Figure 4:**
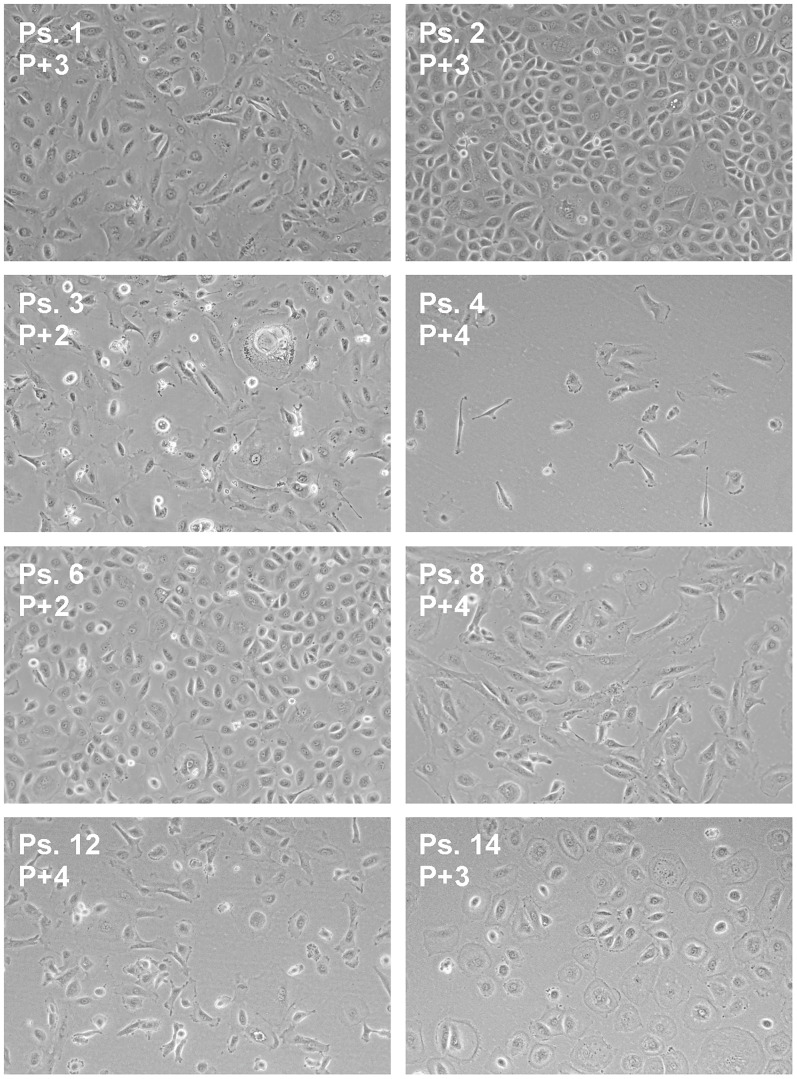
Morphology of ascites-derived adherent cells. Bright field images were captured at indicated passage number (P) at a magnification of 10×.

Ascites is known to contain a mixture of cells, including tumor cells, immune cells, cancer-associated fibroblasts and mesothelial cells [16, 17]. The majority of cells in the unprocessed ascites are immune cells, as determined by CD45-positive staining in flow cytometry experiments. The bulk of the immune cells were removed by overnight adherence of the cells to tissue-culture flasks, during which the bulk of immune cells remains in suspension. After culturing the adhering cells for two to three weeks, expression of the ovarian cancer markers *CA125* and *HE4* was determined by qPCR (Figure 2). Expression levels differed among the samples, but all samples expressed at least one of the two ovarian cancer marker genes. Flow cytometry analysis of five samples (ps. 1, 2, 4, 12 and 14) further confirmed the tumor origin by cell surface staining of the ovarian cancer marker CA125 and the epithelial cell marker epithelial cell adhesion molecule (EpCAM), and intracellular staining of cytokeratin 7 (CK7) (Figure 3). Expression levels were quantified by determination of the ratio of the median fluorescence intensity (MFI) after staining with the specific antibodies relative to staining with isotype control antibody (Table 3). Moreover, the percentage of cells stained positive for the different markers was determined. All samples stained positive for CA125 and CK7. Ps. 12 and ps. 14 also stained positive for EpCAM (Figure 3 and Table 3). The percentage of CA125-positive cells ranged from 42 to 90% and CK7-positive from 80 to 93% (Table 3). Marker expression thus confirmed the EOC origin of the adherent cell fractions. In addition, cells were rather uniform in morphology (Figure 4).

**Table 2:** Detailed characteristics of patients and results of *in vitro* drug sensitivity tests with tumor cells isolated from ascites

Patient		Clinical characteristics			Clinical outcome					*In vitro* outcome		
Ps.	Age	FIGO stage	Type of chemotherapy	Surg.	Outcome surgery	CA125 level at diagnosis, E/mL	CA125 level at end of therapy, E/mL	Platinum sensitivity	BRCA mutation on tumor	GI_50_ carbo, μmol/L	GI_50_ cisplatin, μmol/L	GI_50_ paclitaxel nmol/L
1	71	IIIc	cis/tax^a^	PDS	Optimal	1154	10	Resistant	unknown		2.1	12
2	64	IIIc	carbo/tax	IDS	Complete	1800	34	Resistant	*BRCA1*	43.5		35
3	66	IIIc	cis/carbo/ tax^a^	PDS	Optimal	1242	5	Resistant	*BRCA* negative	80.6	20.6	12
4	64	IIIc	carbo/tax	PDS	Optimal	1481	15	Sensitive	unknown	77.9		10
5	64	IIIc	carbo/tax	PDS	Optimal	2400	26	Sensitive	unknown	90.7		14
6	55	IIIc	cis/tax^a^	PDS	Complete	67	11	Resistant	*BRCA* negative		11.8	45
7	68	IIIc	carbo/tax	None	N/A	3600	258	Resistant	*BRCA* negative	100.0		66
8	58	IVb	carbo/tax	IDS	Optimal	121	15	Resistant	*BRCA* negative	71.4		3
9	64	IVb	carbo/tax	IDS	Complete	1834	35	Sensitive	*BRCA2*	56.8		42
10	52	IVa	carbo/tax	IDS	Complete	1621	15	Sensitive	*BRCA* negative	100.0		35
11	50	IIIc	carbo/tax	IDS	Complete	2500	16	Sensitive	*BRCA1*	84.0		14
12	54	IIIc	carbo/tax	PDS	Optimal	351	14	Sensitive	*BRCA* negative	44.1		24
13	60	IIIc	carbo/tax	PDS	Optimal	500	8	Sensitive	*BRCA* negative	100.0		79
14	51	IVa	carbo/tax	IDS	Complete	3900	9	Sensitive	unknown	31.4		83

^a^Received at least one cycle of intraperitoneal chemotherapy with cisplatin. Abbreviations: Age, age at time of diagnosis; carbo, carboplatin; cis, cisplatin; GI_50_, concentration of 50% cell growth inhibition; IDS, interval debulking surgery; PDS, primary debulking surgery; Ps., patient sample; Surg., surgery; tax, paclitaxel.

**Table 3:** Quantification of cell surface expression of CA125 and EpCAM, and intracellular expression of CK7 in ascites cell samples by determination of the ratio of the median fluorescence intensity (MFI) after staining with anti-CA125, anti-EpCAM or anti-CK7 antibody relative to staining with isotype control antibody

Patient sample	CA125		EpCAM		CK7	
	MFI ratio	% positive	MFI ratio	% positive	MFI ratio	% positive
1	1.35	47	0.93	3.4	10.9	80
2	1.26	42	0.96	1.6	9.65	89
4	1.30	47	0.98	3.3	9.59	85
12	3.01	90	1.41	23	15.5	93
14	2.48	87	1.28	22	6.69	87
SK-OV-3	1.85	47	339	99	1.50	36

Moreover, the percentage of cells stained positive for the different markers was determined. Abbreviations: MFI, median fluorescence intensity.

**Figure 5:**
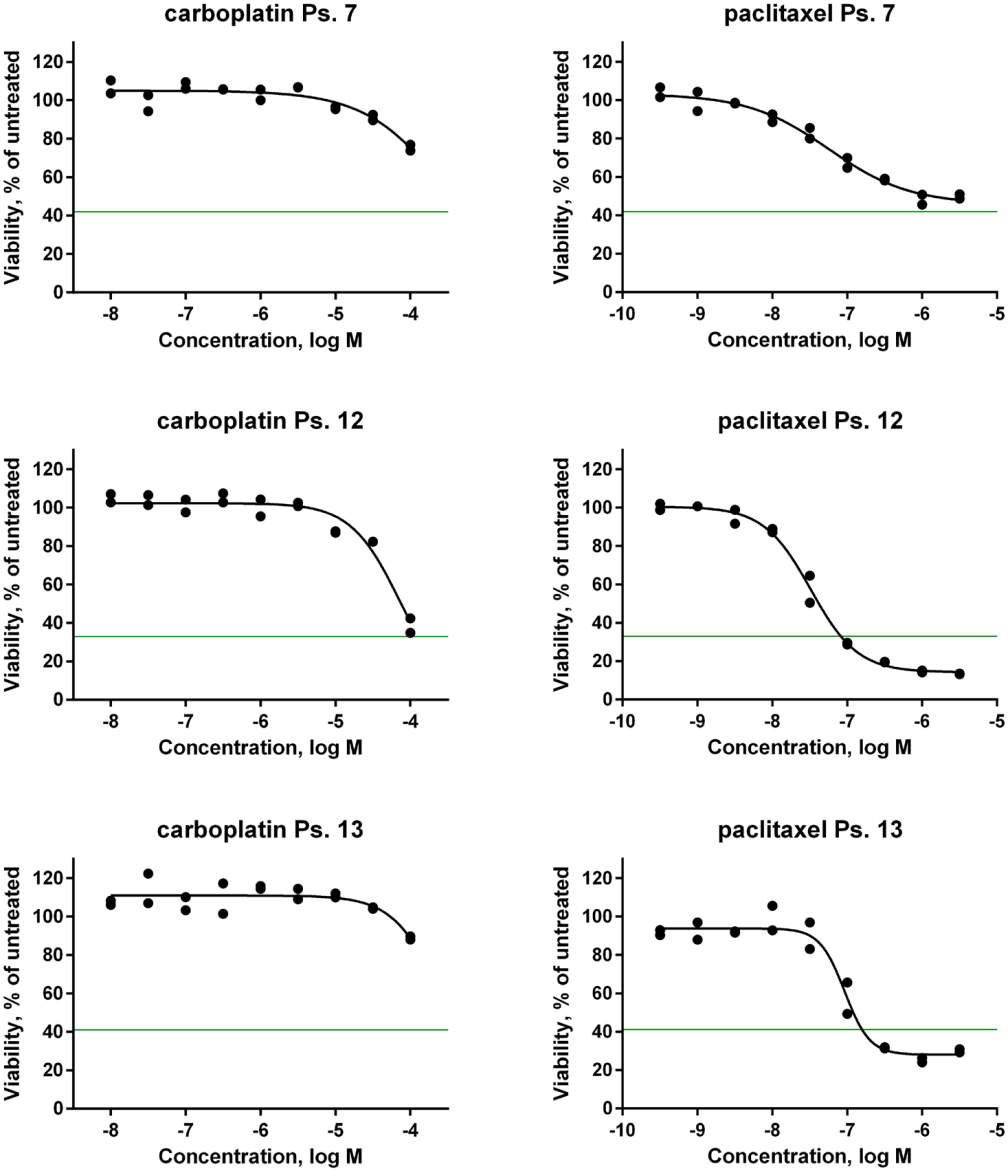
*In vitro* drug sensitivity analysis of primary patient-derived tumor cells. Dose-response curves of the first-line chemotherapeutic agents carboplatin and paclitaxel on tumor cells isolated from ascites of three patients. Cells were seeded in microtiter plates and allowed to adapt for 24 hours before drug was added. Effect on cell growth was determined after 120 hours drug exposure by measuring intracellular ATP content as an indirect readout of cell number. The horizontal green line corresponds to the number of cells before addition of drug.

To determine the sensitivity of the adherent cell cultures to chemotherapy, cell proliferation assays were performed with serial dilutions of the clinical agents (Figure 5 and Supplementary Figure 1). The effect of the chemotherapy on cell growth was quantified by calculation of the drug concentration that resulted in 50% inhibition of cell growth (GI_50_) in comparison to cells treated with vehicle. Clinical data of each patient and results of the proliferation assays are summarized in Table 2. GI_50_ of carboplatin, cisplatin, and paclitaxel were separately correlated with the CA125 level in serum after primary treatment. No significant correlation between CA125 levels and drug sensitivity was observed; carboplatin (Pearson 0.311, *P* = 0.324), cisplatin (Pearson –0.762, *P* = 0.449), and paclitaxel (Pearson 0.333, *P* = 0.245). We then studied the variables classified in groups. The median GI_50_ was 79.3 μmol/L for carboplatin, 11.8 μmol/L for cisplatin and 29.5 nmol/L for paclitaxel. A GI_50_ of carboplatin, cisplatin, and paclitaxel equal to or less their respective median GI_50_ was considered as a ‘good’ *in vitro* response, while GI_50_ values greater than the respective median GI_50_ values was considered as a ‘poor’ *in vitro* response (Table 2). Three patients (ps. 1, 3, and 6) received IP cisplatin, and they all showed a good clinical response. Therefore, only one group of the ordinal variables is available and statistical analyses could not be performed. No significant difference between clinical outcome of CA125 level and GI_50_ of carboplatin (*P* = 0.268), or paclitaxel (*P* = 0.219) could be identified. Also, no significant difference in platinum sensitivity and GI_50_ of carboplatin or paclitaxel was found (both *P* = 1.000), and no difference between *BRCA1* or *BRCA2* mutated samples and the compounds was seen (*P* = 0.213 for carboplatin, and *P* = 0.681 for paclitaxel).

In clinical practice, carboplatin and paclitaxel are given in combination therapy. However, testing of the two agents in mixtures did not increase the response *in vitro* (Supplementary Figure 2 and Supplementary Table 1). In contrast, if the *in vitro* response data to the single agents are combined per case, twelve patients (86%) showed correlation between clinical outcome and the results of the *in vitro* assays. Thus, good clinical outcome correlated with a good response to at least one of the compounds in the *in vitro* assays, and poor clinical outcome correlated to poor *in vitro* response to both compounds. As an example, a poor clinical outcome was seen in ps. 7 (Table 2). After six cycles of chemotherapy treatment, the CA125 level in serum was still elevated (258 E/mL). The patient did not undergo surgery because of the poor biochemical response after neoadjuvant chemotherapy (NACT) and a computed tomography (CT)-scan that still showed considerable tumor mass. The *in vitro* response of the corresponding ascites cell sample was in line with a poor outcome, that is, the GI_50_ was high for both carboplatin (100 μmol/L) and paclitaxel (66 nmol/L) (Table 2 and Figure 5). Another case in which clinical outcome was predicted well is that of ps. 12. The *in vitro* tests predicted a good clinical outcome (Figure 5), which was confirmed by the low CA125 level of 14 E/mL after primary treatment (Table 2). Only two samples (14%; ps. 10 and 13) did not show any correlation (Table 2). Both patients had a good clinical outcome with post-treatment CA125 levels of 15 E/mL and 8 E/mL, respectively, while the *in vitro* response was poor for both compounds. Both had a GI_50_ of 100 μmol/L for carboplatin and, respectively, a GI_50_ of 35 nmol/L and 79 nmol/L for paclitaxel.

## DISCUSSION

In this study, we determined the *in vitro* sensitivity of tumor cells isolated from ascites to first-line chemotherapeutic agents. The results were related to the clinical response of EOC patients to primary treatment. 85% of the samples had a sufficient number of proliferating tumor cells to determine the effect of chemotherapy treatment on cell viability and proliferation. There was no significant difference in sensitivity between the GI_50_ of carboplatin or paclitaxel and clinical outcome. However, when the *in vitro* response to both single agents was considered, twelve patient samples (86%) showed a similar response in *in vitro* drug sensitivity and clinical outcome. In two samples (14%) no correlation was observed.

Although we used a small group of patient samples, this study demonstrates the feasibility of predicting chemotherapy sensitivity by combining the *in vitro* response to the agents, carboplatin, cisplatin and paclitaxel on proliferation of cells isolated from malignant ascites. The use of cells isolated from ascites has a major advantage over the use of surgical biopsies [11, 12], because it is minimal invasive and allows drug sensitivity testing prior to the start of primary chemotherapy treatment. Furthermore, *in vitro* proliferation assays in 2D cell cultures have a major advantage over proliferation assays in 3D or organoid cultures [18], which take much longer and are technically more challenging. In clinical practice, performing *in vitro* testing in ascites prevents exposure of patients that will not respond to toxic chemotherapeutics.

Two samples did not show any correlation. These patients (ps. 10 and 13) had a good clinical outcome, while the cells isolated from ascites of these patient showed low *in vitro* drug sensitivity. Study of the clinical record of one of these patients revealed that the start of adjuvant chemotherapy treatment of ps. 13 was delayed due to a fever of unknown origin. It is known that activation of the immune system can have a positive effect on tumor regression in ovarian cancer [19–21]. Although speculative, this may also have been the case in this patient.

CA125 level as a measure for clinical outcome can be debated. CA125 is elevated in, on average, 80% of EOC patients at time of diagnosis [22]. In our study, one patient did not have elevated CA125 at diagnosis. Therefore, it was not possible to determine clinical outcome and the patient was excluded. Furthermore, only one patient (ps. 7) showed poor clinical outcome with a CA125 above 35 E/ml after primary treatment. However, this is in line with clinical practice where 80–85% of patients respond to primary chemotherapy treatment [5]. Another possibility to measure clinical outcome is to include disease-free or overall survival of patients in the assessment. However, these data were not available for all patients at the time of evaluation of clinical response. Moreover, by using disease-free survival as clinical criterium, other aspects of treatment, such as the outcome of the debulking surgery, also play a role. We evaluated platinum sensitivity as a measure of clinical outcome, but this did not show correlation with CA125 level after primary treatment. However, the definition of platinum resistance is arbitrary, and can be influenced by the timing and modality of follow-up, independent of tumor biology [23]. Since we were mainly interested in the primary response of the ovarian tumors on chemotherapy, we chose CA125 level. This points out the difficulty of determining an appropriate measure for clinical outcome. Therefore, in an expansion of this study, different variables to measure the response to chemotherapy will be taken into account, including CA125 level, outcome of CT-scan using RECIST-criteria [24, 25], outcome of the debulking surgery, and the histopathologic chemotherapy response score [22]. These variables, together with platinum sensitivity, PFS, and OS will further substantiate the conclusion for clinical outcome.

We used strict inclusion criteria to have a homogenous group of ovarian cancer patients. Therefore, patients with tumors with a histology other than high-grade serous were excluded. However, women with mucinous tumors are described as poor responders to chemotherapy, and having poor prognosis [26]. Adding patients with, for instance, this histology type would broaden the clinical applicability of the *in vitro* drug sensitivity tests.

Besides the proliferation assays, the addition of gene mutation analysis of *BRCA* could add more value in predicting chemotherapy response. *BRCA* mutations in the tumor have been linked to improved survival [7]. For three of the ten patients tested for somatic *BRCA* mutation, a *BRCA1* or *BRCA2* mutation was identified. Another marker predicting chemotherapy response is the immune checkpoint programmed death-ligand 1 (PD-L1). High expression of PD-L1 has been linked to poor prognosis [27], although opposite results have been reported as well [28]. In an expansion of the current study, the relationship between *BRCA* mutations, and PD-L1 expression on ascites tumor cells and clinical outcome will be investigated.

In conclusion, our study shows the feasibility of assessing *in vitro* chemotherapy sensitivity on tumor cells isolated from ascites. A larger, prospective study, which takes into account the insights that were gained in this pilot study, is ongoing and will be reported in due course.

## MATERIALS AND METHODS

### Patients

Ascites samples of advanced stage EOC patients were used. The samples were selected from both the Gynecologic Oncology Biobank of the Radboud university medical center and the prospective ASCITES study (since 2018). Ascites was obtained during primary debulking surgery, or during paracentesis for diagnostics or symptom relieve with the aim to use the ascites for research. Three hospitals participate in the ASCITES study: Radboud university medical center, Canisius Wilhelmina Hospital in Nijmegen, and Catharina Hospital in Eindhoven (The Netherlands). The following eligibility criteria were applied: women of eighteen years or older, who were diagnosed with advanced stage high-grade serous ovarian cancer, had a sufficient amount of ascites to collect, which contained enough vital cells, were chemotherapy naive, and signed informed consent. Patients with a history of cancer except basal cell carcinoma, with concurrent malignant disease, or who did not finish primary treatment were excluded. The following data of patients were collected from the electronic patient files and anonymously stored in a database: age, performance status, histology of tumor, CA125 level, *BRCA* mutant status of the tumor, order and type of (chemotherapy) treatment, and follow-up data six months after finishing primary treatment. The study was conducted with approval of the Medical Ethical Review Board of Nijmegen and Arnhem and the local medical ethical committees of Canisius Wilhelmina and Catharina Hospital (no. 2015–2060 and 2018–4528).

### Clinical response

To define clinical response to therapy, the status of remission was determined, using the level of CA125 in serum. The reference range in clinical practice is 0–35 E/mL. Of the patients with an elevated CA125 level at time of diagnosis, a serum CA125 level of 35 E/mL or higher after primary treatment was defined as a ‘poor’ clinical outcome. Clinical outcome was defined as ‘good’ when CA125 was equal or below 35 E/mL. Platinum resistance was defined as the progression during, or recurrence within six months after finishing primary treatment. Patients were labelled platinum sensitive if no signs of recurrent disease occurred within six months after finishing primary treatment.

### Mutation status

The mutation status of the *BRCA1* and *BRCA2* genes in the tumors was determined by DNA sequencing. *BRCA* genetic analysis is performed in all newly diagnosed and recurrent cases of EOC in the Netherlands since 2016. Data on the *BRCA* gene mutation status are lacking for several cases before 2016.

### Tumor cell enrichment and culture

The ascites was filtered, cells were collected by centrifugation and then frozen as described by Wefers *et al.* [29]. Ascites cell samples were enriched for tumor cells by seeding in tissue culture flasks and allowing to adhere overnight. The next day, non-adherent cells were removed and confirmed to contain primarily immune cells by flow cytometry. The adherent cells were cultured for two to three weeks. Cell culture medium was advanced RPMI (Thermo Fisher), supplemented with 10% fetal bovine serum, 1% glutamax and 1% penicillin/streptomycin. Bright field images were captured with a Nikon D3200 camera on an Axiovert 25 CFL light microscope (Zeiss). Brightness and contrast filters were applied on the images. Cells were collected by trypsinization to characterize in gene expression analysis (qPCR), flow cytometry, and proliferation assays.

### Gene expression analysis

RNA was isolated with RNeasy (Qiagen, Venlo, the Netherlands). cDNA was prepared using QuantiTect Reverse Transcription kit (Qiagen). qPCR was performed in a Bio-Rad CFX96 cycler using SYBR™ Select Master Mix (Thermo Fisher). The thermal cycle protocol used was as follows: 50°C for 10 min, 5 min initial denaturation at 95°C, and 40 cycles of 10 seconds denaturation at 94°C, 20 seconds annealing at 60°C, and 30 seconds extension at 72°C. A dissociation curve was added at the end of the cycle. PCR data were analyzed using the 2^-ΔΔCT^ method [30] and both the β-actin (*ACTB*) and ribosomal protein S18 (*RPS18*) genes were used as housekeeping controls. The primers for the qPCR were designed in-house and synthesized at Thermo Fisher. Primers were: HE4 forward: CAAGAGTGCGTCTCGGACAG; HE4 reverse: TTAATGTTCACCTGGGGGCA; CA125 forward: CACAGACAACGTCATGCAGC; CA125 reverse: TGGGAGTTGTAGGAGGCTCA; β-actin forward: CAAGAGATGGCCACGGCTGCTTCCA; β-actin reverse: ATGGAGTTGAAGGTAGTTTCG; ribosomal protein S18 forward: CGATGGGCGGCGGAAAAT; ribosomal protein S18 reverse: CGTTCCACCTCATCCTCAGTG.

### Flow cytometry

For surface staining, cells were harvested and washed in 0.5% (w/v) bovine serum albumin (BSA) in phosphate-buffered saline (PBS), before incubation with labeled antibodies for 30 min. After washing twice with BSA in PBS, 7-aminoactinomycin (Miltenyi Biotec) was added and fluorescence was measured on a Guava easyCyte 12-HT flow cytometer (Luminex). For intracellular staining, cells were fixed in 4% paraformaldehyde for 10 min at 37°C and subsequently permeabilized with 0.1% PBS/Triton X-100 for 15 min at room temperature. After incubation with 10% normal goat serum (Sigma-Aldrich) for 30 min and FcR blocking reagent for 5 min to block aspecific binding, cells were incubated with primary antibody for 30 min, washed with PBS and incubated with secondary antibody for 30 min. After another wash, fluorescence was measured. Flow cytometry data were analyzed and histograms were made with Kaluza Analysis 2.1 software (Beckman Coulter). Cells with negative fluorescence intensities caused by fluorescence compensation were excluded from visual representation, but were included in the quantification of the data. The percentage of cells stained positive for the different markers was determined using the Enhanced Normalized Subtraction method [31]. To ensure tumor cells were studied, and to measure the expression of immune markers, antibodies for the following markers were used: CD45, immune cells (Miltenyi); CA125, ovarian cancer cells (AssayPro); EpCAM (CD326), epithelial cells (Miltenyi); FSP-1, Fibroblast antibody, fibroblasts and epithelial cells (Miltenyi); CK7, epithelial cells (Abcam). The immortalized ovarian cancer cell line SK-OV-3 was used as a control. The cell line was obtained from the American Type Culture Collection (Manassas, VA, USA.).

### Proliferation assays

*In vitro* drug sensitivity was determined in cell proliferation assays after five days incubation with drug. Cells were counted and seeded in 384-well plates for proliferation assays as described [32]. The cell number was optimized to maximize the assay window and to ascertain that growth was not limited by cell density. Carboplatin (MedKoo), cisplatin and paclitaxel (Sigma Aldrich) were stored as dry powders at 4°C. Carboplatin and cisplatin were freshly dissolved in ddH_2_O at the day of the assay. Paclitaxel was dissolved in dimethylsulfoxide (DMSO). Compound effects were measured in a 9-point dilution series in duplicate. As readout in the proliferation assays, intracellular ATP content was determined as an indirect measure of cell number, using ATPlite 1 Step solution (Perkin Elmer, Groningen, The Netherlands). Paclitaxel was tested in a range of 0.32 nmol/L to 3160 nmol/L for all samples. Carboplatin and cisplatin were tested in three ranges: 100 nmol/L to 100 μmol/L, 316 nmol/L to 316 μmol/L, or 1 μmol/L to 1 mmol/L. The same ranges for both compounds were used for the same samples. Exposure time was 120 hours for all compounds. To determine the potential synergistic effects of carboplatin and paclitaxel *in vitro*, 9-point dilution series of one of the two compounds were determined in the presence of the highest inactive concentration of the other compound.

### Dose-response curves

The percentage growth of cells from the ascites fluid was calculated, and the effect of carboplatin, cisplatin, and paclitaxel on cell growth was calculated relative to control wells containing only vehicle. Dose-response curves were fitted by non-linear regression using XLfit5. Maximal and minimal signals were locked, where appropriate, to obtain the best fit as indicated by the *F*-test as implemented in XLfit5. Effect on cell growth was quantified by calculation of the GI_50_, which is the concentration at which the anti-cancer agents inhibited cell growth with 50% in comparison to cells treated with only the vehicle. GI_50_ accounts for starting cell density and differences in proliferation rates between the tumor cell samples [32].

### Statistics

Baseline characteristics were summarized using descriptive statistics. Association between *in vitro* sensitivity tests and clinical outcome were analyzed by Pearson correlation. Normal distribution was confirmed with the Shapiro-Wilk test. For comparison of the clinical outcome and *in vitro* response measurements in ascites binary variables were defined. A cut-off measure for GI_50_ of all compounds (*N* = 14) was determined using the median, for poor or good *in vitro* outcome. Comparisons of clinical outcome, *BRCA* mutations, and the *in vitro* response measurements were assessed with two-sided *T*-test, and the Fisher’s Exact test. Finally, the response to the combination of compounds as it is clinically used was determined. An *in vitro* outcome was considered good if the response to one of the compounds was good, and poor if the *in vitro* tests with both compounds resulted in a high GI_50_. Statistical analyses were performed using IBM SPSS Statistics version 25.

## SUPPLEMENTARY MATERIALS



## Abbreviations

CA125: cancer antigen 125
CK7: cytokeratin 7
CT: computer tomography
EOC: epithelial ovarian cancer
EpCAM: epithelial adhesion molecule
FIGO: International Federation of Gynecology and Obstetrics
MFI: median fluorescence intensity
IV: intravenous
IP: intraperitoneal
PARP-inhibitor: Poly (ADP ribose) polymerase 1 inhibitor
PFS: progression free survival
OS: overall survival
PD-L1: programmed death-ligand 1
ps: patient sample
