# Supplementary material for: Chemotherapy sensitivity testing on ovarian cancer cells isolated from malignant ascites
Source: Oncotarget. 2020 Dec 8;11(49):4570–81. doi: 10.18632/oncotarget.27827 (PMC7733621; doi:10.18632/oncotarget.27827)
Supplement: Supplementary file 1 [file oncotarget-11-4570-s001.pdf]

# Chemotherapy sensitivity testing on ovarian cancer cells isolated from malignant ascites

## SUPPLEMENTARY MATERIALS

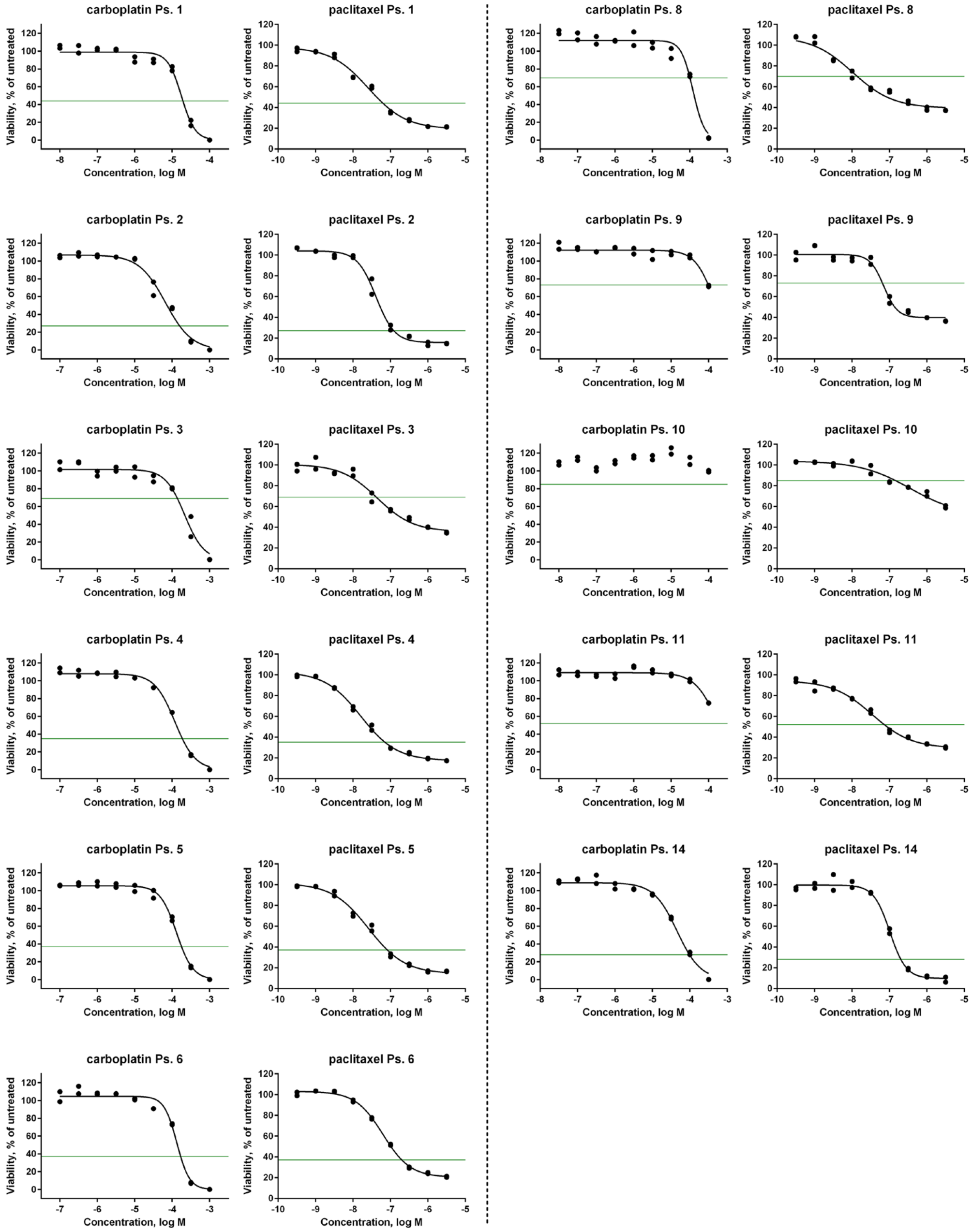


**Supplementary Figure 1: Dose response curves of the first-line chemotherapeutic agents carboplatin and paclitaxel on tumor cells isolated from malignant ascites of ovarian cancer patients.**

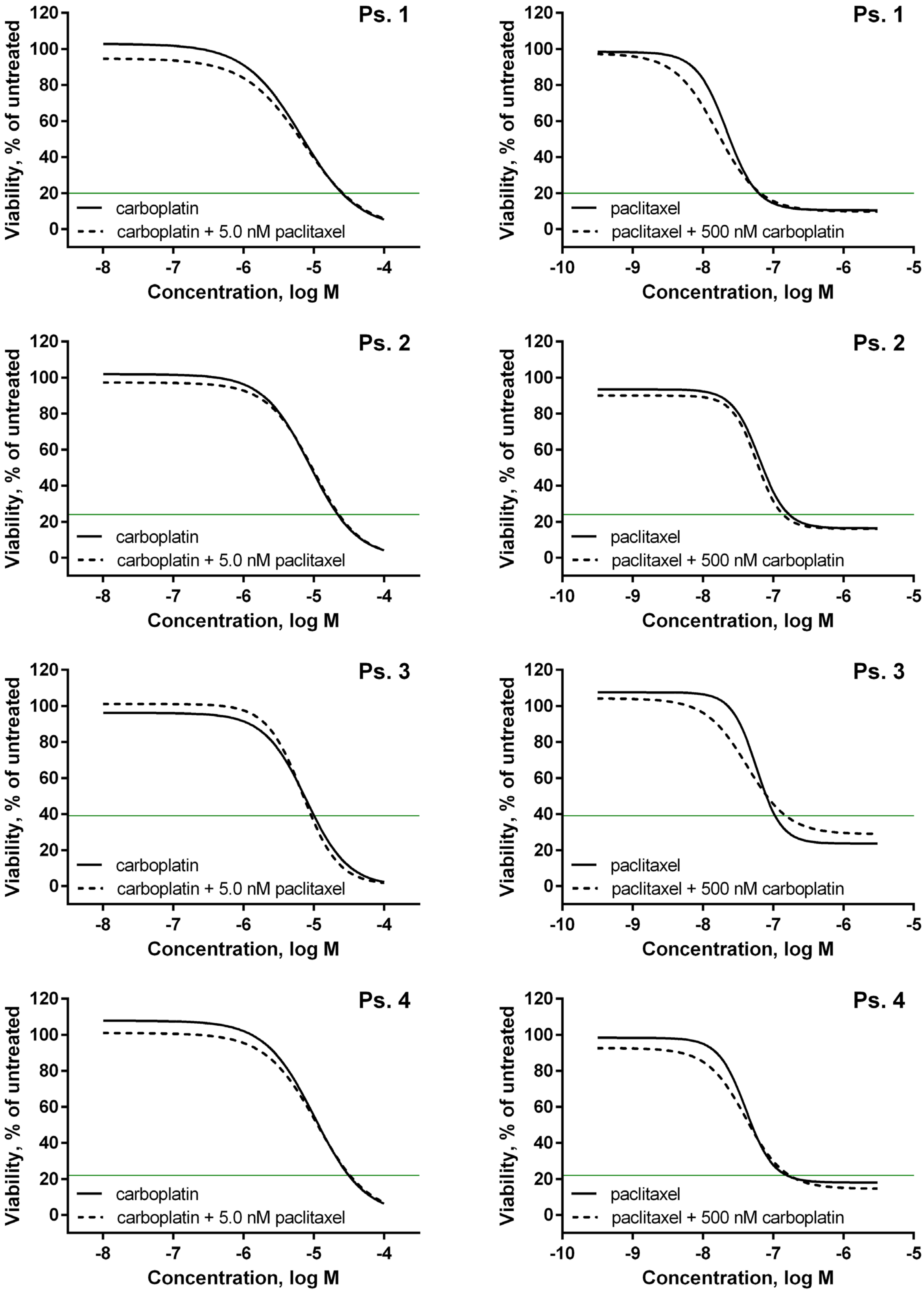
Ps. 1
Viability, % of untreated
Concentration, log M
carboplatin
carboplatin + 5.0 nM paclitaxel
Ps. 1
paclitaxel
paclitaxel + 500 nM carboplatin
Ps. 2
carboplatin
carboplatin + 5.0 nM paclitaxel
Ps. 2
paclitaxel
paclitaxel + 500 nM carboplatin
Ps. 3
carboplatin
carboplatin + 5.0 nM paclitaxel
Ps. 3
paclitaxel
paclitaxel + 500 nM carboplatin
Ps. 4
carboplatin
carboplatin + 5.0 nM paclitaxel
Ps. 4
paclitaxel
paclitaxel + 500 nM carboplatin

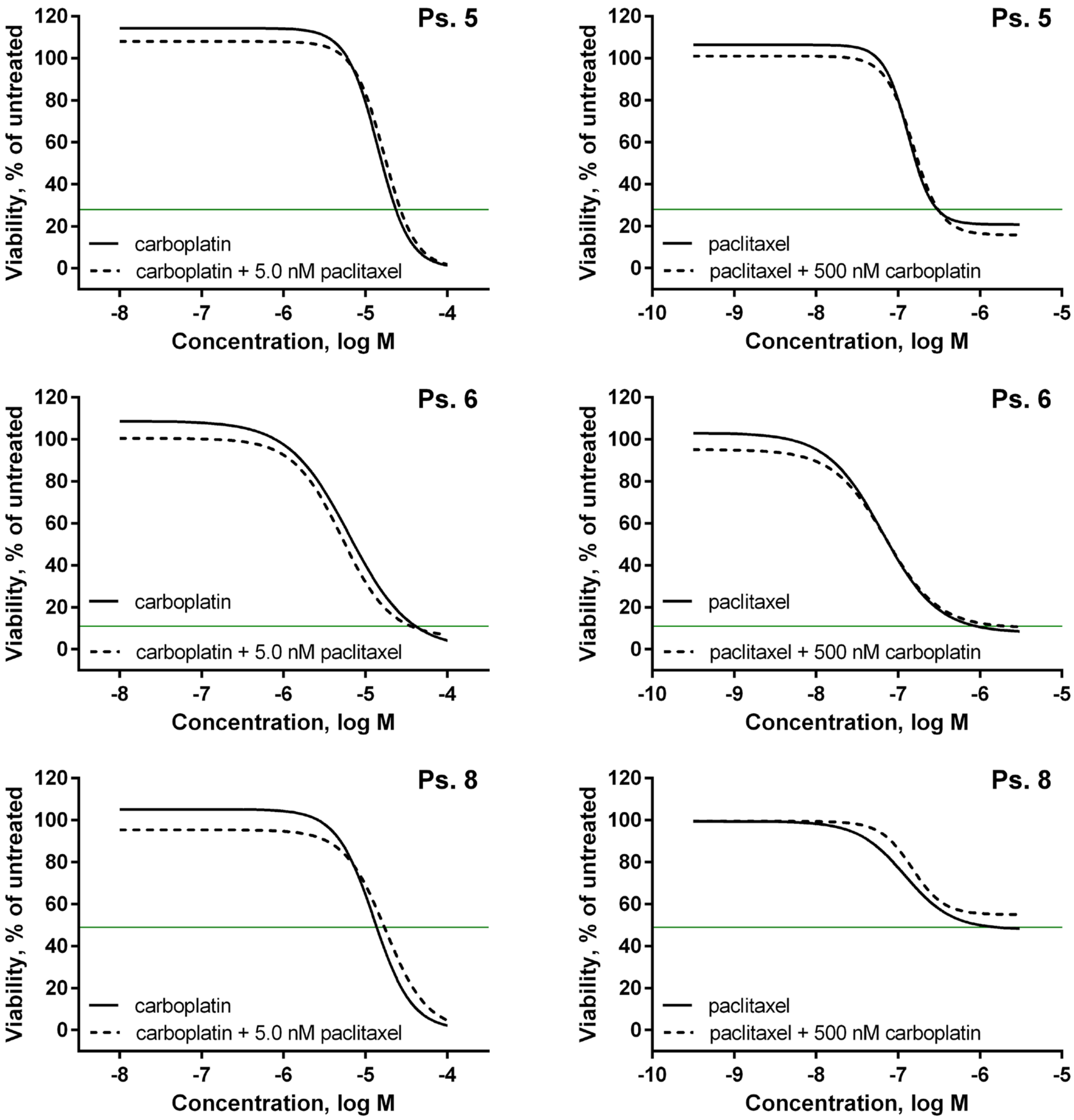


**Supplementary Figure 2: Combination testing of carboplatin and paclitaxel.** Cells of passage 1 were incubated with either carboplatin or paclitaxel at a fixed concentration and a 9-point dilution series of the other compound. Effect on cell growth was determined after 120 hours. $GI_{50}$ values are provided in Supplementary Table 1. It should be noted that a decrease in sensitivity to cisplatin and carboplatin was observed in the proliferation assays with single agents which were performed at passage 2 or 3 (Table 2), in parallel to detailed characterization of ovarian cancer markers by RNA expression analysis and flow cytometry (Figures 2–5).

**Supplementary Table 1: Results of *in vitro* combination drug sensitivity tests with tumor cells isolated from ascites**

| Patient | *In vitro* outcome combination studies | | | |
|---|---|---|---|---|
| Ps. | $GI_{50}$ carboplatin (µmol/L) | $GI_{50}$ carboplatin + 5.0 nmol/L paclitaxel (µmol/L) | $GI_{50}$ paclitaxel (nmol/L) | $GI_{50}$ paclitaxel + 500 nmol/L carboplatin (nmol/L) |
| 1 | 4.6 | 4.8 | 20 | 14 |
| 2 | 6.1 | 6.8 | 58 | 54 |
| 3 | 4.3 | 4.3 | 50 | 35 |
| 4 | 7.4 | 7.6 | 39 | 36 |
| 5 | 11.5 | 13.5 | 125 | 136 |
| 6 | 8.2 | 5.1 | 90 | 103 |
| 8 | 7.5 | 9.1 | 118 | 166 |
